# Inclusive education and related policies in special needs schools in South Africa

**DOI:** 10.4102/ajod.v13i0.1358

**Published:** 2024-09-11

**Authors:** Amukelani P. Mahlaule, Cheryl M.E. McCrindle, Lizeka Napoles

**Affiliations:** 1 School of Health Systems and Public Health, Faculty of Health Sciences, University of Pretoria, Pretoria, South Africa

**Keywords:** special needs schools, learners with disabilities, inclusive education policy, teachers, healthcare workers, South Africa

## Abstract

**Background:**

Post-apartheid, the education system shifted its focus from a segregated education system to an inclusive education system, which resulted in greater consideration of the role and function of special needs schools. In 2014 the National Department of Basic Education developed and implemented an inclusive approach and policies to provide guidelines on the running of special needs schools (SNS). The study was conducted in six SNS in Ekurhuleni South District, South Africa.

**Objectives:**

The study explored the experiences of teachers and healthcare workers when implementing policies in SNS in the study area.

**Method:**

This exploratory qualitative study used purposive sampling to select 13 teachers and healthcare workers for in-depth interviews. Collected data were analysed using inductive thematic analysis and ATLAS-ti version 23.

**Results:**

Teachers and healthcare workers had different working experiences and understandings about inclusive education and policies, as well as their role in implementing these policies. Experienced challenges included lack of training, limited resources, lack of parental support, issues with differentiated curriculum, an unacceptable teaching environment; and poor referral systems. These challenges evoked strategies such as improvising, collaborating, and referring. Participants indicated that they required further training, resources, and support to successfully implement inclusive policies.

**Conclusion:**

Both teachers and healthcare workers agreed that resources were lacking at all SNS represented. Staff training was urgently needed as the current curricula at SNS were differentiated for learners with physical and intellectual disabilities.

**Contribution:**

Findings may inform policy implementation and change in SNS.

## Introduction

An inclusive education system in South Africa takes into consideration the diverse learning needs of all learners (Donohue & Bornman 2014). This is in line with the framework outlined in the Education White Paper 6: Special Needs Education: Building an Inclusive Education and Training Systems (EWP6). This policy has made provisions for improving the role and functioning of schools within the system including mainstream, full-service, and special needs schools (SNS) (Department of Basic Education 2001). Special needs schools are designed to provide appropriate support for low, medium, and high level of needs of learners (Directorate Inclusive Education (2014). Special schools exist to offer maximum support to school children experiencing barriers to learning (Khumalo & Hodgson 2017). Despite efforts and progress made to make inclusive education a reality, disability remains an overlooked issue in education especially in developing countries (Mizunoya, Mitra & Yamasaki 2018).

The type of disability and level of support required determines the type of special school required by learners with special needs in South Africa (Directorate Inclusive Education 2014). Disability type includes but is not limited to physical disability, intellectual disability, hearing and vision impairment (Directorate Inclusive Education 2014). Children with disability experience barriers to learning, such as inaccessible curricula, an unsupportive teaching environment, as well as difficulties with understanding the language and strategies used (Department of Basic Education 2001). These barriers to learning often include a lack of access to curricula, difficulties with language, and lack of parental support (Manicka Naicker 2018).

An inclusive model of education must be implemented to ensure that barriers to learning are addressed for all learners; however, disability should remain high on the agenda (Makoelle 2012). Education policies were developed and implemented to convert the South African education system into a more inclusive one (Engelbrecht et al. 2016). This was in support of Sustainable Development Goal (SDG) 4, Target 4.5: improving access to quality and inclusive education and ensuring that those who are vulnerable, including people with disabilities are not discriminated against and are able to access all levels of education (Franco & Derbyshire 2020).

Understanding the link between learning and health yields better academic and health outcomes (Hunt 2015). It is evident that health issues can negatively affect learners’ academic performance. For example, learners with health difficulties are likely to attain poor grades, not because they are not academically able, but because of health problems not being addressed (Hunt 2015). Disability should not be seen as a problem that hinders education attainment; therefore, systems should be put in place to support learners with special needs (Hayes & Bulat 2017). This enables policies for SNS such as EWP6, the National Strategy on Screening, Identification, and Support (SIAS); the Integrated School Health policy (ISHP) should adequately address the needs of learners requiring extra support (McKenzie 2021).

The EWP6 has established principles and guidelines to promote inclusive education. This policy further highlights the government’s plan for special schools and aims to implement inclusivity and improve quality education and service delivery through infrastructure development and resource mobilisation (Department of Basic Education 2001). The SIAS provides a blueprint for identification and evaluation of learners as well as provision of relevant intervention to learners experiencing barriers to learning; including those with disabilities (Department of Basic Education 2014). On the other hand, ISHP serves as an integral part of the development of inclusive education by focusing on provision of health services in schools and helps to address health-related issues and minimise barriers to learning (DOH & DOBE 2012). This act involves the collaboration of the Department of Health, Department of Basic Education and Social Development, to ensure that all schools have access to health services.

Policy should always be designed with implementation in mind, as this would determine the progress of the designed policy (Pollack Porter, Rutkow & Mcginty 2018). The success in executing this policy relies on the implementation process (Hudson, Hunter & Peckham 2019). Implementation of the policy at a local level should be performed by the Provincial districts and aligned with both National and Provincial goals (Mokitimi, Schneider & De Vries 2018). However, policy documents are generally designed with ambiguous objectives and lack implementation guidelines thus leading to unsuccessful achievement of the intended goals (Engelbrecht et al. 2016). For example, EWP6 suggests that the implementation of an inclusive education policy is cost effective. However, ambiguity is seen with regard to availability of funds so as to address issues related to infrastructure and mobilising of human resources (Donohue & Bornman 2014).

The lack of policy clarity has presented challenges to successful implementation. A study was conducted by Adewumi and Mosito (2019) at Fort Beaufort district primary schools in the Eastern Cape province of South Africa to explore the experiences of teachers in implementing inclusive education for learners with special needs. It reported challenges such as unclear curriculum guidelines, little support for teachers, as well as a lack of funding as the driving forces preventing implementation of inclusive education (Adewumi & Mosito 2019). Another South African study explored the South African schools’ role in the implementation of the integrated health approach in the Emfuleni District Municipality in Gauteng province (Kwatubana & Kheswa 2014). Kwatubana and Kheswa (2014) suggested that the Integrated Health Approach seemed to be ineffective because of unspecified persons being responsible for implementing it in public schools. The study further highlighted the fact that there needed to be more support or involvement, between the School Based Support Team (SBST) from the National Department of Basic Education and parents, regarding learners requiring health services. Additionally, a lack of allocated resources was shown to be a limiting factor in providing learners with professional healthcare services in all schools (Kwatubana & Kheswa 2014).

In the City of Tshwane, in Gauteng province, Ramukumba, Rasesemola and Matshoge (2019) found that compliance to the ISHP was poor, because of inconsistent service delivery and a lack of participation from learners and relevant partners to ensure that this programme succeed (Ramukumba et al. 2019). Furthermore, Zembe-Mkabile (2021) highlighted that the coronavirus disease 2019 (COVID-19) pandemic made it difficult for schools to offer certain ISHP because of the pandemic restrictions that were put in place in 2020. Difficulties encountered could be attributed to unreadiness of departments and infrastructure, as the schools had to comply with legislated COVID-19 regulations. This resulted in schools prioritising only those activities that were relevant for teaching and learning, which affected the delivery of the health services stipulated in ISHP guidelines (Zembe-Mkabile 2021). A study conducted by Ntseto et al. (2021) reported that implementing the Policy on (SIAS) was challenging because of teachers not being well trained for teaching learners with special education needs, who often lacked the motivation needed to implement the policy (Ntseto et al. 2021). A study conducted by Hess (2020) on teachers’ perceptions around implementation of SIAS in mainstream schools reported that the curriculum was rigid, so it was difficult to reach and teach all learners according to their level of needs, which impacted on access to quality education (Hess 2020). According to Hayes and Bulat (2017), teachers in low- and middle-income countries reported that being unable to adapt the curriculum made it difficult to meet the educational needs of learners with disabilities.

Despite these challenges, there are schools where inclusive education is implemented successfully and is accessible to all learners with special needs (Walton 2011). A study conducted in Fort Beaufort District primary schools highlighted the fact that support from managers, and the ability of teachers to accommodate and improvise, led to the successful implementation of inclusive education to learners with special needs (Adewumi & Mosito 2019). In addition, the implementation of the National School Nutrition Programme that is aligned to the South African Department of Basic Education has proven successful, because of effective collaborative efforts and involvement of different corporate sponsors (Zembe-Mkabile 2021).

Taking into account challenges and achievements in implementing school health policy, it is important to evaluate and monitor the implementation process to identify barriers (Hunt 2015). It has been recommended that issues arising from the implementation process should be addressed by improving the support system, provision of resources, and strengthening the involvement of different stakeholders (Hunt 2015). Successful implementation of the ISHP involves collaboration of team members; including relevant departments, parents, and learners. A policy with clear guidelines and strategies will enable a smooth implementation process and could achieve the target goals for the intended programme thus improving learner health and academic performance (Ramukumba et al. 2019).

These studies conducted in South Africa, mainly explain the experiences of teachers implementing inclusive policies in mainstream schools. To the best of our knowledge, there remains little investigation performed in schools for learners with special needs in the study area. Therefore, this study explored, determined, and identified the understanding, challenges, strategies, and support in inclusive policy implementation by teachers and healthcare workers in the area under study.

## Theoretical framework

This study uses the bottom-up approach for implementing policy as a theoretical framework promulgated by different authors including Berman (1978); Hjern and Porter (1981); Hjern (1982); Hjern and Hull (1982); Hull and Hjern (1987); and Lipsky (1978) (Stofile 2008). The bottom-up approach looks beyond central planning. ‘For successful implementation, experiences of ‘on the ground’ implementers need to be taken into consideration as they can provide factors affecting implementation of policies, which can form a basis for policy change (Stofile 2008). The goals and strategies of the policy must be aligned with both policy implementers and the target groups (Girdwood 2013). The top-down approach to policy implementation emphasises the importance of policy clarity and directives by policy developers to ensure that the relevant goals are met (Donohue & Bornman 2014). This study takes the bottom-up theoretical orientation to implementing policy and argues that relevant policy goals can be met if daily experiences of teachers and healthcare workers as implementers are considered. This could include understanding the causes of policy failure as observed on the ground. Their discretion at delivering policy shifts from a ‘one size fits all’ policy to a suitable context, could lead to success in policy implementation (Donohue & Bornman 2014). Progress with policy implementation could be made if what is happening on the ground is studied and analysed.

## Research methods and design

### Study design

The study used an exploratory qualitative design to assess the implementation of inclusive policies by teachers and healthcare workers in SNS in Ekurhuleni South District. In this study, the exploratory qualitative approach enabled the researcher to gain a clear insight of the phenomenon under study and allowed participants to answer questions based on their personal and professional point of view, and experiences when implementing inclusive policies (Hammarberg, Kirkman & De Lacey 2016). Qualitative research uses the scope of gathering people’s personal understanding and opinions about concepts (Taylor, Bogdan & Devault 2015). The rationale for adopting a qualitative approach is motivated by the need to comprehend the experiences of teachers and healthcare workers in implementing inclusive policies in SNS, and further gain a deeper perspective of their daily challenges and coping strategies.

### Study setting

The study was conducted in SNS in Ekurhuleni South District, Gauteng Province, South Africa. Ekurhuleni District is situated to the east of the City of Johannesburg. It falls under the Ekurhuleni Metropolitan Municipality with a population of approximately 3 379 104 as recorded in 2016 (Cooperative Governance and Traditional Affairs 2020). There are two main school districts, namely Ekurhuleni North and Ekurhuleni South, which together include approximately 420 schools. This study was conducted in six SNS located in Ekurhuleni South District (Department of Basic Education 2013). These SNS offer a specialised curriculum addressing learner needs for those with physical disabilities, intellectual disabilities, and those who are hard of hearing (Department of Basic Education 2014).

### Study population and sampling method

Teachers and healthcare workers (*n* = 13) were recruited from these six SNS in the study area to participate in the study, with the selection criteria that they had been employed for a period of 2 years or more in SNS. This study used a purposive non-randomised sampling method, which allowed the researcher to gather data from participants regarding their experiences on inclusive policy implementation (Etikan, Musa & Alkassim 2016).

Guest, Bunce and Johnson (2006) reported that a saturation level of 92% in qualitative research can be reached with 12 in-depth interviews. When there are no further dimensions on the problem, the data are considered to have reached a saturation level (Vasileiou et al. 2018). Data saturation in this study was reached with 13 participants.

### Data collection

In-depth interviews (*N* = 13) were conducted face to face in English. They consisted of open-ended questions listed in an interview guide that had been approved by the University Ethics Committee (Ethical Clearance Certificate number: 538/2022). Each interview consisted of a series of open-ended questions related to understanding, challenges and strategies and support required with inclusive policy implementation at SNS. Only two participants agreed to audio recordings. The others (*N* = 11) were recorded manually in writing. Interviews were conducted by the principal researcher (First author), between 14:00 and 16:00 in the office of the principal at each SNS. Each in-depth interview lasted approximately 30 to 40 min.

### Data analysis

This study used thematic analysis and data were coded in accordance with questions answering the study objectives. Themes and patterns were observed, organised, categorised, and conclusions were drawn (Braun & Clarke 2006). This process follows the steps or phases outlined for thematic induction: including familiarisation with data, coding, generating and reviewing themes, as well as defining and naming themes (Herzog, Handke & Hitters 2019). Quotes were also used for emerging and uncategorised themes (McMillan & Schumacher 2010). The qualitative software analysis programme, ATLAS.ti version 23.2.1 (2023) was used for open coding of data during the analysis process, for storage and management, safe keeping, as well as manual analysis and interpretation (Friese, Soratto & Pires 2018).

### Ethical considerations

The study received ethical clearance from the Faculty of Health Sciences research committee, University of Pretoria with approval number 538/2022, and Gauteng Department of Basic Education, with approval number 2022/387. Written consent was obtained from the District Educational Office, as well as the principal, and participants in each participating school. Confidentiality of data was maintained by coding responses anonymously. Only the researcher had access to the data that are kept in a secured database. Personal identifiers such as names of participants and names of the schools were removed from the research documents and assigned with key codes to ensure that participants remained anonymous.

## Results

### Demographics

Table 1 shows demographic information of participants (*N* = 13) representing the six SNS in the study area. Participants represented more than one profession: for example, healthcare worker and teacher.

**TABLE 1:** Demographics of participants.

Demographic profile	Frequency
**Age range**	
25–30 years	1
31–40 years	5
41–50 years	2
50–60 years	5
**Profession**	
Teachers:	7
Healthcare workers:	6
• Professional nurse	1
• Speech therapist	2
• Occupational therapist	3
**Number of years employed**	
1–10	6
11–20	3
21–30	3
31–40	1
**Highest level of qualification**	
Diploma	1
BA Honours degree	10
Masters	2
**Type of special school**	
Physical disability	8
Intellectual disability	5

### Experiences of working in a special needs school

The teachers and healthcare workers described their experiences at SNS as challenging, ‘frustrating’, ‘interesting’, and/or ‘exciting’. Most participants reported that working in a special school had been challenging and frustrating because of lack of a specific curriculum for learners with disabilities as they have to constantly review lesson plans. One of the participants indicated that moving from a mainstream school to the SNS was very frustrating because she had to review her lesson plans, which was not easy; she said:

‘I have experience working in a mainstream school for foundation phase learners, so coming here for the first time…it was really frustrating because I just went to class expecting to teach and learners will just look at you and not understand what you mean or what you are writing. It was the first time I found out the whole class has a problem understanding. So, I had to review my lesson plans and there was no DCAPS (Differentiated Curriculum and Assessment Policy Statement) at that time, so it was not easy for me.’ (Participant 3, teacher, age group 51–60 years)

On the other hand, one participant reported that it was difficult at first; however, with time he learnt how to proceed and he is now managing to work effectively in the SNS. One healthcare worker indicated that it was an interesting experience for her as she came from the Department of Health to an environment where children had different disabilities and learning needs. She commented:

‘I am a nurse that loves her job, the environment was interesting, and I stayed here because of my passion in these 8 years.’ (Participant 1, healthcare worker, age group 51–60 years)

### Admission criteria in special needs school

All participants reported several uncertainties about the admission criteria. The type of disability that ought to be catered for by their own SNS was not necessarily observed but rather other disabilities were included in the admission criteria. For example, participants from SNS for learners with physical disabilities, and/or mild intellectual disabilities, showed concerns that learners with other disabilities such as autism, and profound intellectual disability are currently admitted, whereas the school does not cater for them. One participant said:

‘It was supposed to be for physically challenged but currently I feel like it caters for both physical and nonphysical because we have few learners that have severe intellectual disability (SID) and profound intellectual disability (PID).’ (Participant 8, healthcare worker, age group 25–30 years)

### Inclusive policies: Understanding of inclusive education

Participants explained their understanding of inclusive education based on inclusive policies. Most participants mentioned that their understanding was that inclusive policies were about providing learning opportunities to learners; despite their challenges; and that they should get the same education as learners in mainstream schools with no limitations. One participant said that her understanding of inclusive education is based on the EWP6:

‘Education White Paper 6 caters for all learners, as a school we need to be seen supporting them because our constitution says every child has a right to learn so it gives the opportunity to learners regardless of the challenge. Its emphasis is on their strength and that we must support their skills.’ (Participant 5, teacher, age group 51–60 years)

Another participant described EWP6 as an inclusive policy that addressed the imbalances of the past and made a great impact:

‘It was to address the imbalances of the past; politics played a role there because learners were not getting education based on the disability, so they had to make changes for every learner to have access, irrespective of their disabilities.’ (Participant 6, teacher, age group 51–50 years)

Some of the participants based their understanding of inclusive education on the processes to be followed during the implementation of the SIAS policy, which includes screening, identification, assessment, and support. Of which, most of the participants indicated that they knew what the SIAS policy is but could not explain it as they did not fully understand it. Only two participants showed their understanding of what the SIAS policy was for SNS. One of the participants showed his understanding of the SIAS based on the benefits it brought for the school. He reported that since implementation of the SIAS policy, there were no incorrect placements of learners in the school:

‘With SIAS, we as the Department we are benefiting big time. We are no longer look at a child with face and say this learner should be at special school or not. The psychological assessment report helps the department for proper placement.’ (Participant 6, teacher, age group 41–50 years)

Only two participants based their understanding of inclusive education on ISHP; however, they could not explain fully what it entails. Two participants commented as follows:

‘I think with integrated school health policy is inclusive in a sense that as LSEN or rather special schools, we need to be seen working with the Health Department. Is a collaboration part of it; for example, if a child needs to be referred, they can be seen by a specialized person from health?.’ (Participant 5, teacher, age group 51–60 years)

‘I am aware of such policy although I am not sure of the contents of the policy. But I think it means the therapists and teachers work together to assist the learners.’ (Participant 6, teacher, age group 41–50 years)

Some of the participants (Seven participants) based their understanding of inclusive education on their role in implementing inclusive policies. They explained that their role was to implement inclusive education and policies and to ensure that support is given to learners in need. One participant said:

‘My role is to make sure that the policies achieve their objectives: I implement the strategies as a teacher.’ (Participant 3, teacher, age group 51–60 years)

### Challenges with policy implementation

Six key aspects were identified from the policies to illustrate the challenges experienced when implementing inclusive policies. The key factors needed for implementation included: differentiated curricula, training opportunities, limited resources, health education and promotion, provision of health services, school environment, and type of disability in a learner.

#### Differentiated curriculum

Two ideas emerged when it came to the differentiated curriculum: the time factor and lesson planning. Specifically, DCAPS looks at equipping learners with the necessary knowledge and skills for self-fulfilment and participation in the community. Four teachers revealed reporting time as a serious challenge when it came to the differentiated curriculum. They highlighted that they did not think that the policy makers took into consideration the timetable when designing the curriculum for SNS learners. They reported that having to plan lessons for each learner was time consuming:

‘They want us to do things their way not children’s way; and it’s a lot of work for the learners.’ (Participant 7, teacher, age group 51–60 years)

‘The curriculum says we need to teach them 20% theory and 80% skills, and I need to teach them 3 subjects a day; of which is a problem in one day.’ (Participant 3, teacher, age group 51–60 years)

They further reported challenges with adapting lesson plans to suit the needs of each learner, as learners with different disabilities and learning barriers were included in the same class. What exacerbated the issue was not having assistant teachers or class aids. One of the participants emphasised that the standard of education changed when they adapted the curriculum because of unclear guidelines on the adaptation process:

‘The challenge is with the lesson plans; we need to plan for individual learner and differentiate for each and to be honest the standards of the education we give drops. The level of functioning of the learners affects the quality of education we give.’ (Participant 2, teacher, age group 50–60 years)

#### Training opportunities

Respondents were unsure about their roles. Certain participants received training on the policies under study, namely, EWP6, SIAS, ISHP, while others did not receive training. How they accessed training was also different. They also felt there were questions about the impact that lack of training had on implementation of policy. Table 2 illustrates the findings and the number of times a code (key word or phrase) was used during the interview. The higher number shows how frequently the code was used.

**TABLE 2:** A summary of training findings (*N* = 13).

Code group	Codes	Frequency (*N* = 13)
Received training	Yes	04
	No	06
	Not sure (It was long time ago)	03
Who should provide training	Personal responsibility	04
	Department must provide	10
	I don’t know	03

As indicated in Table 2, only four participants received training on SIAS policy and two on EWP6. None of the participants reported having received training on ISHP. Participants who reported that they had not received training indicated that lack of training affects how they support the learners. Without training, they are not aware or sure of what is expected from them. Instead, they end up doing what they think is right. One of the participants reported that she struggled in class because she was not trained for teaching at a SNS and it was a challenge.

On the other hand, participants who received training (*N* = 6) indicated that it had helped them in knowing how to go about supporting the needs of the learners and that guidelines in the policies show exactly what needs to be done. Of those participants who received training, only two received training on EWP6; four on SIAS, and no participant received training on ISHP. There was however, one participant who reported that she received training on SIAS policy and still found the training not effective. She said:

‘Yes, only SIAS but I feel like it was not effective because it was done in one day. Most examples they were making during the training were not relevant to our school.’ (Participant 10, healthcare worker, age group 31–40 years)

Another participant who reported having received training opined:

‘The training was vague; this train a trainee is not working. It seems like the people who are doing the trainings they themselves do not understand the policies. It affects everything in implementation. I think people who draw up these policies must do the trainings.’ (Participant 13, healthcare worker, age group 31–40 years)

Participants who reported having received training a long time ago felt that the lack of continuous training and being updated on the revised policies affected both knowledge and understanding about implementing these policies.

#### Limited resources

Human resources and assistive devices were revealed as limited at these schools. The majority of participants (*N* = 7) reported that there was a shortage of physical human power to assist both in the classroom and with therapy. Healthcare professionals (therapists) reported that they are limited in schools and the demand to meet learner needs is high. This was supported by one of the school principals when he said the following:

‘We don’t have enough therapists. The posts are there but it is difficult to fill them or to retain current therapists we have due to incentives.’ (Participant 4, teacher, age group 41–50 years)

On the other hand, teachers reported that being the only one in class, with no class assistance is challenging, when they prepare lesson plans and focus on individual learner needs. Another participant reported with frustration that they do not have a social worker in the school, yet the learners have psychosocial issues and they do not know what to do in such a situation.

The availability of expensive assistive devices and the lack of information on how to use the devices effectively was also seen as problematic. Healthcare workers reported that the assistive devices they are supposed to use are expensive, but schools, sponsors, and parents often end up buying cheaper ones with poor quality, which affects delivery of therapy. Another challenge mentioned, mostly by teachers, is that they do not know how to use the assistive devices as they have no background in health sciences. One participant said:

‘We do struggle with assistive devices. We do have the money and we can and we do buy them, but we do not know how to use them.’ (Participant 4, teacher, age group 41–50 years)

#### Health education and promotion

Most of the participants (*n* = 11) reported to be fine with health promotion and education in the school because of working collaboratively as a team of teachers and healthcare workers. A minority of participants (2 out of 13) reported having challenges with promoting good hygiene with the learners because of little to no involvement of parents. Another challenge reported was that there were no proper referring channels for learners and this affects interventions that the learner is meant to receive From this, two challenges emerged, namely a lack of parental involvement and poor referral channels.

#### Provision of health services

Five healthcare workers were interviewed and they reported a lack of resources, space restrictions, inconsistent skills offered to learners in the school, a lack of parental involvement and not having enough time and enough therapists to cover all learners in the school as factors that affected the provision of effective support. Two participants from two different schools reported that they needed more resources to support learners that initially were not catered for by the schools. They further explained this challenge in the following words:

‘We do not have resources to treat SID learners. We end up taking from our pockets to get the little resources we need for a learner. We have a learner with autism, and we do not have the resources to support the learner.’ (Participant 8, healthcare worker, age group 25–30 years)

Three of the five participants who were occupational therapists showed great concern around the level of available skills in the school. One participant said:

‘Skills, if I was to rate it, I would give it zero. It is so sad for our learners; the skills are in the classroom. Let me give you an example, there is a salon class and learners are placed there, but there are learners that cannot do beauty things, but they are forced to do it by just being in that class. Then it’s needle work but because you are placed in that room you are forced to do needle work.’ (Participant 11, healthcare worker, age group 31–40 years)

One of the participants said the following about the frustration with space restrictions:

‘I will talk about space restrictions. As speech therapists in the school, we don’t have an optimal treatment room, we are giving therapy in a “computer lab” which is also our office space. We get to alternate when we see learners and we don’t do group therapy at all. The whole space thing is challenging and affects how we give effective therapy, the environment is not conducive.’ (Participant 13, healthcare worker, age group 31–40 years)

#### The school environment

Almost all (*n* = 12) of the participants indicated that the SNS where they worked could support learners and that it was accessible to learners. Some of the participants (*n* = 4) however reported challenges with maintenance of the infrastructure and revealed that newly revamped toilets and showers were not suitable for learners with physical disabilities. One participant reported that the school is not accessible for any learner with physical disabilities:

‘We do have 3 or 4 learners with physical disabilities, and we don’t have a disabled toilets to cater for them. We must assist the learners to the toilet. The toilet is so small, and we have to go inside the toilet with the learner to make sure they don’t fall inside. Another challenge is that toilets are far from the classes. It is good for learners with SID not for the physical disabled.’ (Participant 11, health care worker, age group 31–40 years)

One participant had concerns about the safety of the school as it is built on dolomite land, which can cause sinkholes, putting everyone in the school at risk:

‘The only challenge I have is that the school is built on dolomite land, we are getting unsafe every day. We have learners and hostel workers that are left in the school every day on land that is not safe. This is a known problem for years, but nothing has been done, they must do something very fast.’ (Participant 13, health care worker, age group 31–40 years)

#### Disability type as a factor to implementation

**FIGURE 1:**
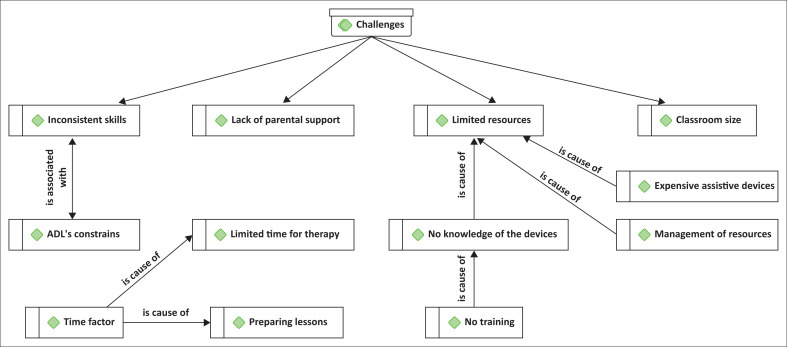
Challenges with inclusive policy implementation.

Most of the participants (*n* = 7) indicated that disability of the learners does not affect the implementation process of policies, as provision is made for them in the schools where they are placed. On the other hand, some participants (*n* = 5) reported that inclusion becomes a challenge when there are wrongly placed learners as they cannot offer optimal support. Figure 1 shows an ATLAS-ti graphic illustration that is a summary of challenges.

### Alternative strategies employed

**FIGURE 2:**
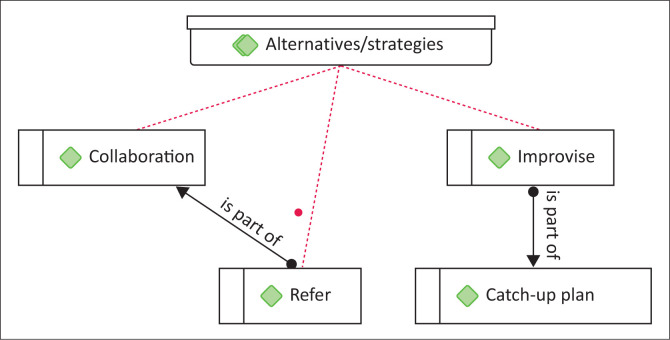
Alternative strategies.

During the analysis of the interviews, three alternative strategies emerged. These were collaboration, improvisation and referral as alternatives to resolve the challenges faced by participants. Figure 2 is an ATLAS.ti graphic illustration of the three themes.

As illustrated in Figure 2, the majority of participants (*N* = 8) indicated collaboration within the school is a support mechanism that has been beneficial to achieving the intended goals of the policies. They further indicated that despite having limited resources staff (both teachers and health workers) improvise with the curriculum and therapy resources to ensure successful implementation of the policies. One of the teachers opined:

‘A catch plan assist. If they did not finish the work, they do it first the following day before the subjects for that day starts.’ (Participant 7, teacher, age group 31–40 years)

Another participant said the following in response to an alternative to providing health services despite the challenges:

‘I am forced to improvise, and work with what I have because at the end of the day I must deliver.’ (Participant 13, healthcare worker, age group 31–40 years)

### Recommendation and support required

Seven ideas for improving support for learners emerged from the analysis. These were: communication and transparency, in-service training, training on policies, correct placement of learners, qualified personnel for special schools, more human resources, and more skills support for the learners.

One participant said the following about the support they need from the Department of Basic Education:

‘For the department to provide training regularly, and to hire people that have qualifications on inclusive education for special needs schools.’ (Participant 4, teacher, age group 41–50 years)

Another participant, a teacher, said the following:

‘The Department is always supporting us, but we have therapists in the school so in-service training to understand their roles and how they can help us. Actually, everyone working in the school, must be developed on how to support the learners.’ (Participant 5, teacher, age group 50–60 years)

One participant reported that they need support from the Department of Basic Education to help with developing the curriculum at her school:

‘We need to implement a different ATP for special schools, not same as the ordinary schools; because we cannot have work similar to mainstream. We cannot rush lessons with our learners.’ (Participant 9, teacher, age group 31–40)

## Discussion

Teachers and healthcare workers found working in SNS to be challenging and interesting. They demonstrated an understanding of inclusive education and related policies in SNS; however, there were uncertainties around some of the policies and what was expected of them, which could affect the implementation process. Ramukumba et al. (2019) highlighted the importance of a policy with clear guidelines and strategies for successful implementation. Thus, teachers and healthcare workers’ uncertainties about the policies might have a negative impact on how inclusive policies are implemented and could hinder achievement of target goals. Challenges with differentiated curricula, specifically the time factor and difficulties with lesson planning, expand on the findings from other studies (Hayes & Bulat 2017; Hess 2020).

In a study conducted by Kwatubana and Kheswa (2024), limited resources were reported as a challenge to providing health services at school. Therefore, findings from this study support the fact that the lack of resources including human resources and assistive devices are indeed a hindrance to providing effective health services at SNS. This was worsened by the lack of appropriate training opportunities for the existing limited staff. Staff were either qualified as teachers or health workers. While the SNS needed personnel who could fill both these niches, further specialised training for both categories of staff was essential but lacking in practice. These results indicated that lack of specialised training in policies made the implementation process challenging. This confirms findings from a study conducted by Ntseto et al. (2021) on challenges experienced by teachers in special education, as well as a study by Zembe-Mkabile (2021).

A lack of parental involvement and poor referral channels were also reported as challenges. The same findings were found by a study conducted by Ramukumba et al. (2019) in the City of Tshwane related to compliance to the ISHP, which highlighted a lack of participation from relevant partners such as the Department of Health and the Department of Basic Education, teachers, healthcare workers, learners, parents, and the community. The ISHP stipulates that learners should receive Primary Health Care services in the schools and where such services are not available, mechanisms should be in place to ensure that learners have access to these services.

To ensure successful implementation of policies at SNS, more training is required on the integration of the skills of healthcare workers, teachers, and parents, as well as improving the school infrastructure. These include better universal design of schools to meet the needs of those scholars who attend SNS as well as increasing human and material resources.

In spite of the challenges faced by professionals at SNS, teachers and healthcare workers have found strategies to surmount these difficulties such as the use of improvising and collaborating with relevant stakeholders. In essence, teachers and healthcare workers act according to their convictions by improvising to meet learner needs leading to successful implementation of inclusive education (Hunt 2015). These are employed as a result of the lack of training and resources with regard to how they should implement relevant policies. It is therefore difficult for both teachers and healthcare workers because their challenges are at distinctive and varied levels of the system. The efforts made by teachers and healthcare workers are an indication of complex issues, which require complex solutions.

### Limitations

The study acknowledges the limitations that probe for a possible further investigation. This study had 13 participants from the area under study, which is a representative of a minority of the schools’ population. Therefore, the results cannot be generalised to a larger population. Similar research studies in other areas are therefore required and recommended to enable a clear understanding of the experiences of teachers and healthcare workers in a larger scale. The strength of the study is that the respondents were not limited to specific questions; issues arising from the interviews were explored as they emerged.

### Recommendations

This study provides insightful information on issues relating to policy implementation in SNS. Based on the findings of this study, there is a need for both the Department of Basic Education and Department of Health to review SNS and existing policies as the majority of the respondents felt that policy implementation came short, although they understood what was meant by ‘Inclusive education and related policies’. The following can be done when reviewing these policies:

- Assess the current state of SNS to determine whether they are still serving the main objective of meeting the unique needs of learners with disabilities.
- Review the current curriculum offered in SNS to ensure that the curriculum offered at the schools is aligned with the educational needs of learners.
- Take into consideration issues with policy implementation to inform policy’ review and amendment where applicable. The department should stay up to date with latest research to identify gaps in policy implementation.
- Allocate adequate and effective resources and funds to enable implementation of inclusive education.
- Offer maximum support to basic implementers of the policies. This should be inclusive of compulsory training of teachers and healthcare workers on policies and provision of continuous professional development.
- Integrate the roles of parents, teachers, and healthcare workers to optimise education outcomes for children with special education educational needs by ensuring active participation and training of all stakeholders.

## Conclusion

The study revealed the experiences of a selected group of teachers and healthcare workers in implementing policies in SNS of Ekurhuleni South District, South Africa. The results outlined here are intended to better understand issues related to policy implementation experienced by both teachers and healthcare workers. The results of the study showed that working in SNS has challenges, including a lack of training opportunities on policies, limited resources, and support, as well as issues with the curriculum and referral system. As the bottom-up theory suggests, progress can be made in realising and implementing inclusive education and related policies in SNS; should the experiences of teachers and healthcare workers working in SNS in Ekurhuleni South District be taken into consideration as the study’s findings highlighted possible factors that affect the implementation process. Precisely, this can form the basis for change in the policy-implementation process for achieving inclusive education for all.
